# Progress in Medicine

**Published:** 1898-08-13

**Authors:** 


### Progress in Medicine.

### FEVERS.

**Typhoid.**—It is a startling fact that the Army Medical Returns for 1896 show that one-third of all the mortality in the British Army was due to this disease.[1] More than four-fifths of the deaths occurred in India, the Straits Settlements showed a remarkable immunity, while Egypt and Bermuda had recently rates actually higher than India, though that for Bermuda was reduced in 1896 by sanitary measures. This astonishing mortality from a preventable disease deserves most serious attention, and it is to be hoped that improved management in India, where a special investigation is being made, will succeed in removing a serious blot on the administration. The vitality of the typhoid bacillus in the soil when fæcal or other organic matter is present, has been shown to be far greater than was supposed. Robertson[2] has been able to isolate them 315 days after they were placed in the ground, even after the frost and rain of winter, provided that the place was watered from time to time with solutions of organic matter. It would seem that a low temperature hinders their multiplication, but when warm weather returns they increase rapidly. These results are confirmed by Sidney Martin, who found the bacilli throve for at least 105 days in garden mould which was kept warm and moist, though they perished in pure, unmanured soil. Other specimens were isolated by Professor Delépine[3] from earth between the bricks of a midden, but the duration of their sojourn there is not certain. Finally, it appears that the Munich records[4] show that a considerable amount of infection may be due not to infected water supplies, but to contaminated subsoil, for the death-rate, which had reached 1,490 per million, fell to 190 and 180 when the soil was purified, while the subsequent introduction of a pure water supply did not greatly alter the mortality, which regularly rises with the fall of the ground water. Thus, though water-borne typhoid is the most common cause of epidemics in this country, we must not lose sight of occasional infection from a polluted subsoil which has become the home of vast numbers of bacilli, and from which the dust and household utensils may be in turn infected.

**Treatment.**—J. Michell Clarke[5] allows only a fluid diet—milk, beef tea, barley water, and the yolk of eggs, with alcohol only when there is heart failure, quiet delirium, or insomnia, and in the apyrexial period before solid food can be given. With regard to antipyretics he points out that the use of the common drugs to reduce the temperature has not lowered the mortality below 12 to 16 per cent., while the statistics of an enormous number of cases treated by cold baths show a death-rate of only 6 to 8 per cent. Moreover, this method does not merely lower the temperature, but it also causes a great elimination of the toxin of the disease. The writer accordingly uses cold baths for all except the mildest cases, that is, for all whose temperature reaches to more than 102 deg. or 103 deg. Cold sponging suffices for mild attacks, but for the others he claims that baths have a greater and more prolonged effect; that the temperature can always be reduced by them, though sponging may fail; that their effect on the vascular and nervous systems is more marked, the pulse becoming fuller and slower; and, finally, that they do not involve more movement of the patient if sufficient nurses are at hand to lift him in and out of the bath. The use of calomel for two or three nights during the first week of fever is of advantage, and may be followed by the administration of hydro or beta naphthol every three hours day and night. The bismuth-beta-naphthol seems the most satisfactory, and is nearly tasteless; it checks diarrhœa, reduces the number of organisms in the intestines, and prevents distension, but it is difficult, after all, to estimate the real effect of these intestinal antiseptics on the mortality. Dreschfield, indeed, gives some figures which point to a saving of 3 per cent of lives. For hæmorrhage, ice, morphia,' and turpentine are useful, and for sudden cardiac failure injections of caffeine and strychnia. Clarke reports a mortality of only 7·1 per cent. on 70 cases. In the discussion of this paper Shingleton Smith and Markham Skerritt, while acknowledging the value of cold baths, spoke of the use of cold sponging and phenacetin in mild cases, and occasionally of a large dose of quinine, 30-40 grains. Hare and Holder[6] argue that the saving of life by the cold bath treatment is only about 3 per cent., that relapses and hæmorrhage are more frequent, and perforation not rarer when it is employed. They too recommend calomel in the earliest stages of the disease, cold sponging, and only when this fails should the bath be employed. Moderately active massage is also advocated by them, and more nourishment than is usually given to typhoid patients. Alcohol should be carefully administered if the heart begins to fail. In support of their view of the inadvisability of the routine use of baths the editor of the *American Journal of the Medical Sciences* mentions the low mortality in the Maidstone epidemic, where only 7 5 per cent. of the patients died out of a total of 1,885 under all kinds of treatment. It should, however, be noted, that single epidemics vary greatly in virulence, and that the statistics referred to by Clarke show that the average mortality for England is still 17 per cent., while those which relate to the cold bath treatment are drawn from the records of many epidemics in several countries, and give a death-rate of only 6 to 9 per cent. among many thousands of patients. L. Weber[7] advises minute repeated doses of calomel, or even a single large one, and the washing out of the colon by a copious enema when treatment is begun. For food he allows peptonised milk and farinaceous soups only until the temperature has been normal for a week. Cold sponging is employed every three hours as long as the temperature is high, though where the conveniences for baths are present better results can be obtained. In the Alfred Hospital, Sydney, R. S. Skirving[8] allows his patients a carefully prepared diet to alleviate the monotony of milk and lime water. This includes "prepared" milk (with lime and barley water), well boiled sago and custard, jellies, chocolate creams, tea, cocoa, and coffee, meat juices and beef tea, lemon drinks, egg flip, and oysters with the beards removed. He employs Burney Yeo's acid mixture, with cold sponging and baths for hyperpyrexia only. His mortality appears to have been 17 per cent. in 500 cases. Much discussion continues in America on the Woodbridge treatment, which consists of the constant administration of antiseptics, with copious draughts of cold water throughout the disease. The Woodbridge treatment has been reported on favourably by some physicians, and the author claims that he can cut short the disease if treatment is commenced early enough. Dudley Palmer,[9] in defending the possibility of aborting the disease, argues that it begins with a local sepsis, and that systemic infection is later. Hence, if we can attack the point where the first germs multiply, we may put an end to the disease; but Manges replies that cases are found without any apparent intestinal lesion at all, as in the instance lately published at the Johns Hopkins Hospital.

[1] Lancet, Feb. 26, [2] Lancet, Jan. 22, [3] Lancet, Feb. 26. [4] Lancet, Feb. 5. [5] Brit. Med. Chir. Jour., Mar. [6] Amer. Jour. Med. Sci. [7] Post Graduate, April. [8] Australian Med. Gaz., Dec. 20. [9] Med. Rec., Feb. 19.

